# A comprehensive and systematic review on resveratrol supplementation as a promising candidate for the retinal disease: a focus on mechanisms of action from preclinical studies

**DOI:** 10.3389/fphar.2025.1615910

**Published:** 2025-07-11

**Authors:** Xiao-Min Lv, Na Li, Lin-Wei Chen, Cheng Sun

**Affiliations:** ^1^ Department of Pharmacy, The Affiliated Taizhou People’s Hospital of Nanjing Medical University, Taizhou, China; ^2^ Department of Pharmacy, Affiliated Hospital of Nanjing University of Chinese Medicine, Nanjing, China

**Keywords:** resveratrol, retinal disease, animal models, systematic review, mechanisms

## Abstract

**Background:**

Resveratrol is a natural polyphenolic compound that shows great potential in neuroprotection, anti-inflammation,and antioxidation. Previous studies have demonstrated that resveratrol can effectively treat various animal models of retinal diseases.

**Purpose:**

The aim of the research was to use an animal experimental model to assess the effectiveness of resveratrol in treating retinal-related diseases in various animal models of retinal diseases such as ischemia-reperfusion injury, diabetic retinopathy, glaucoma, chronic ocular hypertension, optic neuritis, age-related macular degeneration, and retinopathy of prematurity. Furthermore, this study aims to reveal the underlying mechanisms of resveratrol related to the treatment of retina-related diseases.

**Methods:**

A search was conducted across several databases, including PubMed, EMBASE, the Cochrane Central Register of Controlled Trials, Web of Science, and OVID. The search time was from the establishment of the database to October 2024 to collect studies on resveratrol intervention in animal models of retinal diseases. The studies included in this paper adopted the SYRCLE’s risk of bias tool. Stata 16.0 and RevMan 5.4 software were used to analyze and visualize the results.

**Results:**

Our meta-analysis comprises 26 studies and 365 animals demonstrates the following effects of resveratrol compared to the control group: a significant increase in the number of retinal ganglion cells (SMD = 3.91, 95% Cl = [2.97, 4.86], *p <* 0.00001) and superoxide dismutase activity (SMD = 3.14, 95% Cl = [0.96, 5.33], *p =* 0.005). Moreover, a decrease in malondialdehyde (SMD = −9.29,95% Cl = [−12.84, −5.74], *p <* 0.00001), reactive oxygen species level (SMD = −4.29,95% Cl = [-6.25, −2.32], *p <* 0.0001), cyclooxygenase-2 (SMD = −2.66, 95% Cl = [−4.01, −1.30], *p =*0.0001), tumour necrosis factor-α(SMD = −3.96,95% Cl = [−6.27, −1.65], *p* = 0.0008) and interleukin-6 (SMD = −3.32,95% Cl = [−4.20, −2.44], *p <* 0.00001) was observed. The A-wave amplitude and B-wave amplitude showed an increase respectively (MD = 105.92,95% Cl = [58.99, 152.84], *p <* 0.00001); (MD = 158.00,95% Cl = [86.35, 229.65], *p <* 0.0001), along with an increase in inner retinal thickness (SMD = 6.33, 95% CI = [5.10, 7.56], *p <* 0.00001) and total retinal thickness (SMD = 2.70, 95%Cl = [0.77, 4.83], *p =* 0.01). Subgroup analysis showed that different doses of resveratrol were associated with an increase in the number of RGCs (*p <* 0.05). Resveratrol improves retinal diseases through multiple mechanisms: i) Neuroprotection: it activates the SIRT1/NF-κB and Nrf2 pathways, inhibits Caspase-3 expression, and promotes the survival of RGCs and ii) Antioxidation: it upregulates SOD activity, reduces the levels of MDA and ROS, and alleviates oxidative damage and iii) Anti-inflammation: it inhibits the COX-2, TNF-α, IL-6, and NF-κB pathways, alleviating the inflammatory response. These mechanisms resulted in enhanced amplitude of A/B waves, improved retinal thickness and visual function.

**Conclusion:**

Resveratrol has neuroprotective, anti-inflammatory and antioxidant effects through multiple mechanisms, thereby reducing retinal damage and maintaining the structure and function of the retina. This provides preclinical support for its possible therapeutic uses in the management of retinal diseases.

**Systematic Review Registration:**

https://www.crd.york.ac.uk/PROSPERO/myprospero.

## 1 Introduction

The retina, a vital tissue in the human eye, plays a crucial role in light perception and the transmission of visual signals. However, due to inflammatory responses, oxidative stress and neurovascular dysfunction, retinopathy ensues and led to the occurrence of various retinal diseases (Wang et al., 2022). In ischemia-reperfusion (I/R) injury, the recovery of blood flow after ischemia produces reactive oxygen species (ROS) which intensifies the apoptosis of ganglion cells and endothelial cells (Qin et al., 2022). Diabetic retinopathy (DR) is characterized by persistent hyperglycemia that induces mitochondrial dysfunction, generates continuous oxidative stress, promotes the production of cytokines (such as VEGF and IL-6), and leads to retinal neurodegeneration (Yue et al., 2022). Glaucoma and chronic ocular hypertension (COH) are mainly related to elevated intraocular pressure, causing gradual ischemia of the retina and damaging retinal ganglion cells and optic nerve axons (Sim et al., 2022). Age-related macular degeneration (AMD) is caused by degenerative damage to the macula, leading to dysfunction of the retinal pigment epithelium and neovascularization, resulting in irreversible vision impairment. Optic neuritis, characterized by optic nerve inflammation, frequently associates with autoimmune disorders like multiple sclerosis, disrupting signal transmission between the retina and brain (Petzold et al., 2017). Retinopathy of prematurity (ROP) is caused by the retina of premature infants being exposed to a hyperoxic environment after birth, resulting in oxidative reactions and abnormal development of retinal blood vessels (Dammann et al., 2023). Due to the limited treatment methods, the incidence of various retinal diseases continues to rise, imposing a significant burden on both individual quality of life and global healthcare systems (Fisher, 2021). This emphasizes the importance of exploring new treatment strategies.

Resveratrol (RSV) is a naturally occurring polyphenol and the main bioactive compound in red wine, which has been widely studied at present. Beyond red wine, resveratrol is abundantly present in various plants, including grapes (particularly the skin and seeds), peanuts, berries, mulberries, and blueberries (Tian and Liu, 2020). Studies have demonstrated that resveratrol, as a flavonoid drug, has anti-cancer, anti-inflammatory, anti-hypertensive, anti-thrombotic, and anti-addictive effects (Yunusoğlu et al., 2025). Regarding its pharmacological effects in retinal diseases, it also shows multiple targets and has received more attention. The inflammatory response in retinopathy is manifested as the overexpression of various inflammatory factors, such as tumor necrosis factor-α (TNF-α), interleukin-6 (IL-6), and cyclooxygenase-2 (COX-2). These factors can cause damage and death to retinal cells. Resveratrol can significantly reduce the expression levels of inflammatory factors and inhibit the activation of inflammatory signaling pathways (Kovoor et al., 2022). Resveratrol effectively inhibits oxidative stress responses and protects retinal cells from oxidative damage by enhancing the activity of superoxide dismutase (SOD) and reducing the levels of malondialdehyde (MDA) and reactive oxygen species (ROS) (Dziedziak et al., 2021). The damage and death of retinal ganglion cells (RGCs) are among the key factors causing vision loss due to retinal diseases. Resveratrol can significantly increase the number of RGCs and promote survival and functional recovery by activating the neurotrophic factor signaling pathway. Furthermore, resveratrol can enhance the structure and function of the retina as well as its electrical function (Liu et al., 2017).

Despite numerous studies that have explored the role of resveratrol, different researchers have focused on different retinal diseases and studied different indicator outcomes. So far, no researchers have conducted a meta-analysis of the ability of resveratrol to treat retinal diseases. This study aims to systematically investigate the feasibility and potential of resveratrol as a therapeutic agent for retinal diseases through animal experiments. Additionally, it endeavors to comprehensively summarize the various mechanisms by which resveratrol exerts its effects on retinal diseases, promote the translation of animal experimental findings into clinical applications, and provide novel treatment strategies.

## 2 Materials and methods

### 2.1 Protocol and registration

This study was based on a meta-analysis (PRISMA 2020). The detailed procedures and analysis results are listed in Supplementary Table 2. In addition, the study protocol for this review has been registered with the International Prospective Register of Systematic Reviews (PROSPERO) under the registration number CRD42025621096.

### 2.2 Search strategy

In this study, researchers conducted a comprehensive search across five electronic databases (PubMed, EMBASE, Cochrane Central Register of Controlled Trials, Web of Science, and OVID) from the establishment of the database to October 2024. The search strategy included both subject terms and free terms. Two authors (XM Lv and N Li) used the subject terms: “resveratrol”, and “retina” (Supplementary Table 1). The detailed search strategy is shown in Table 1 (PubMed is provided as an example).

**TABLE 1:** Search strategy on PubMed.

#1	Resveratrol [MeSH terms]
#2	(resveratrol) OR (3,4′,5-Stilbenetriol) OR (3,5,4′-Trihydroxystilbene) OR (3,4′,5-Trihydroxystilbene) OR (trans-Resveratrol) OR (trans Resveratrol) OR (Resveratrol-3-sulfate) OR (Resveratrol 3 sulfate) OR (SRT 501) OR (SRT-501) OR (SRT501) OR (cis-Resveratrol) OR (cis Resveratrol) OR (Resveratrol, (Z)-) OR (trans-Resveratrol-3-O-sulfate) OR (trans Resveratrol 3 O sulfate)
#3	(#1) OR (#2)
#4	Retina [MeSH Terms]
#5	(retina) OR (Ora Serrata)
#6	(#4) OR (#5)
#8	#3 AND #6

### 2.3 Inclusion and exclusion criteria

The studies included in this analysis met the following criteria: (1) evaluation of resveratrol effects on an animal models; (2) the intervention group was given resveratrol, and the dose, route or treatment time were not limited; (3) randomized controlled trials; (4) assessment of outcome measures including the number of RGCs, SOD, MDA, ROS,TNF-α, IL-6, COX-2,the inner retinal thickness, the total retinal thickness, the A-wave and B-wave amplitudes.

The studies excluded based on the following criteria: (1) non-rat or non-mouse animal species; (2) include other interventions or drugs; (3) research forms: clinical case reports, reviews and other non-randomized controlled trials.

### 2.4 Study selection and data extraction

Two researchers (XM Lv and N Li) conducted the literature search and data extraction of this study respectively. Disagreements were resolved through discussion with a third party (LW Chen). Duplicate content was removed using EndNote 20 software. Titles and abstracts of the literature were screened to exclude irrelevant studies. Subsequently, a thorough evaluation against the exclusion criteria was performed to identify articles meeting the criteria. Data extracted included the following: (1) baseline content of included studies; (2) rat characteristics including sex, species, age, body weight, and sample size; (3) interventions in the treatment and control groups; (4) study outcomes. Outcome indicator data were reported as mean ± standard deviation. In cases where data were solely presented in graphical format, Engauge Digitizer commercial software was utilized to extract the data from the charts.

### 2.5 Assessment of risk of bias in individual studies

This study was evaluated by the SYRCLE’s risk of bias tool. Two authors (XM Lv and N Li) analyzed seven items in the assessment tool, including randomized sequence generation, allocation concealment, blinding of participants and personnel, blinding of outcome assessments, incomplete outcome data, selective reporting, and bias from other sources.

### 2.6 Statistical analysis

In this study, RevMan 5.4 and Stata 16.0 software were used for data analysis. The effect values selected were standardized mean difference (SMD) and mean difference (MD) based on the result units, with a 95% confidence interval (CI) utilized. In cases of minimal heterogeneity (*p* > 0.1, *I* ^*2*^ *<* 50%), a fixed-effect model was applied for statistical combination. Conversely, in instances of substantial heterogeneity (*p ≤* 0.1, *I* ^*2*^ ≥ 50%), a random-effect model was employed. Forest map was used to analyze the combined effect values of each index. Subgroup analysis was conducted on the experimental groups with varying doses of resveratrol intervention, and sensitivity analysis was carried out to confirm the meta-analysis’s stability. Egger’s test and funnel plots were used to analyze potential publication bias in the results.

## 3 Results

### 3.1 Study selection

**FIGURE 1:**
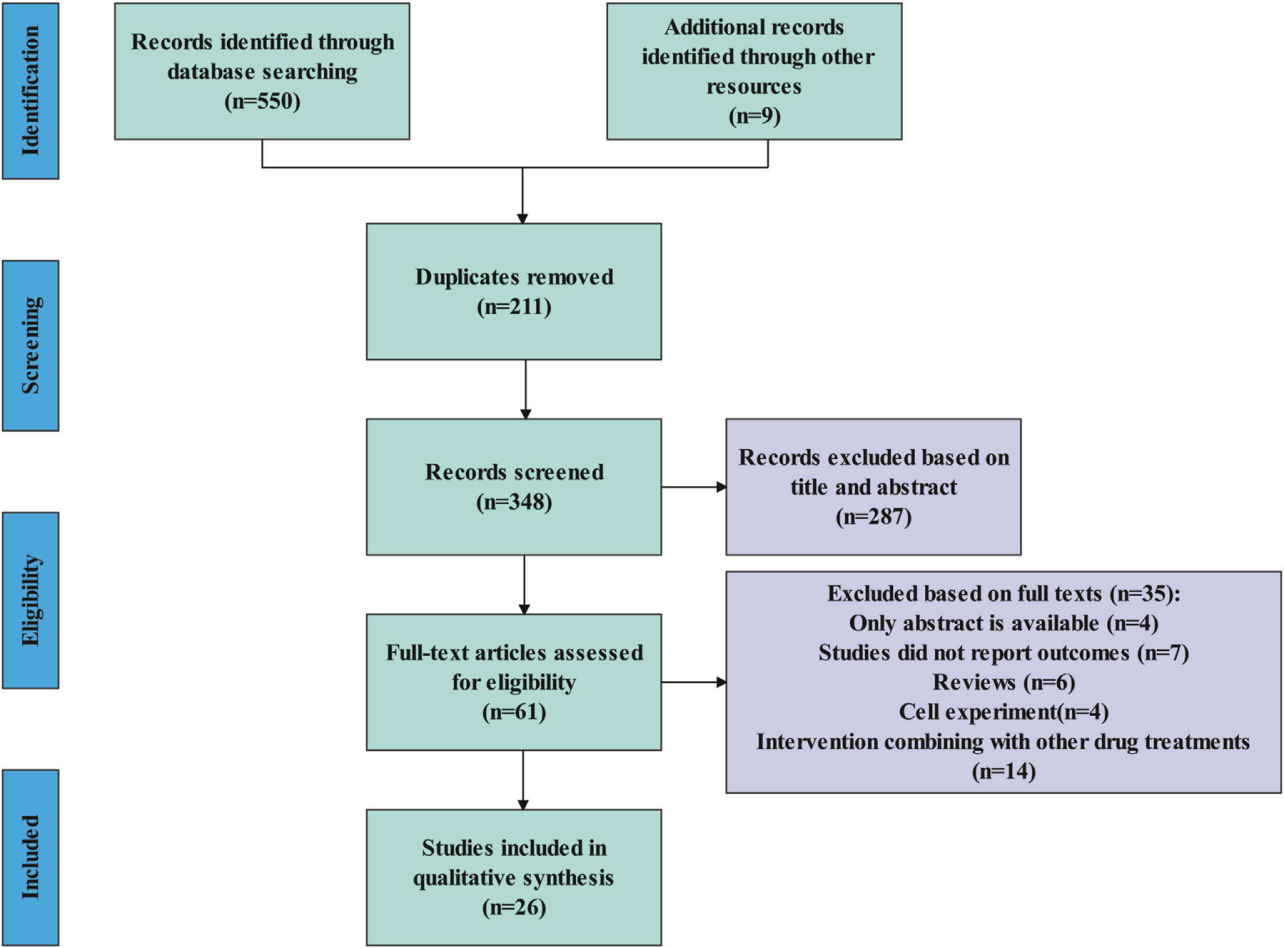
Flow diagram of literature selection.

A total of 559 articles were identified. Using EndNote 20 software, 211 duplicates were removed. Following this, a review of the titles and abstracts led to the exclusion of 285 articles. Upon full-text review, an additional 37 articles were eliminated. Finally, 26 articles were included in the meta-analysis (Figure 1).

### 3.2 Characteristics of the included studies

This meta-analysis included 26 articles, involving 10 studies with Sprague-Dawley rats, 11 with C57BL/6J mice, 4 with Wistar rats, and 1 with Brown Norway rats. The control group consisted of 183 animals, while the experimental group included 182 animals. This study encompasses animal models of retinal injury, comprising 8 models of I/R, 8 models of DR, 3 models of COH, 4 models of glaucoma, 1 model of optic neuritis,1 model of AMD and 1 model of ROP. The number of RGCs was assessed as an outcome in 18 studies, retinal thickness in 8 studies, A-wave and B-wave amplitude in 5 studies, anti-inflammatory markers in 8 studies and antioxidant markers in 8 studies. The characteristics of the studies included in Table 2.

**TABLE 2:** The characteristics of the included studies.

Study	Country	Animals	Weight (g)	Sample size (n)	Type of retinal disease	Treatment group	Control group	Outcome
Yuan et al. (2024)	China	Male C57BL/6J mice (8 weeks old)	25–30	T:5 C:5	DR	RSV 10 mg/kg/d, OG, 1month	No treatment	RGCs↑, MDA↓ SOD↑ Total retinal thickness↑
Shamsher et al. (2024)	United Kingdom	Female C57BL/6J mice (6 weeks old)	NM	T: 5 C: 6	Optic neuritis	RSV 8.44 mg/kg/d, intranasal, 30d	Drug solvent	RGCs↑
Ji et al. (2024)	China	C57BL/6J mice (6–8 weeks old)	NM	T: 4 C: 4	Glaucoma	RSV 20 mg/kg/d, IP, 5d	No treatment	RGCs↑, A-Wave↑ B-Wave↑, IL-6↓
Xiao et al. (2023)	China	Male C57BL/6J mice (8 weeks old)	NM	T: 8 C: 8	DR	RSV 10 mg/kg/d, OG, 12weeks	No treatment	RGCs↑
Chronopoulos et al. (2023)	Germany	Male C57Bl/6J mice (5–6 months old)	NM	T: 8 C: 8	I/R	RSV 30 mg/kg/d, OG, 7d	Drug solvent	RGCs↑, ROS↓
Zeng et al. (2021)	China	SD rats (14weeks old)	180 ± 20	T: 17 C: 17	DR	RSV 10 mg/kg/d, OG, 36weeks	No treatment	RGCs↑
Seong et al. (2021)	Korea	Male C57BL⁄6J mice (8 weeks old)	20–25	T: 4 C: 4	I/R	RSV 20 mg/kg/d, IP, 5d	No treatment	RGCs↑, ROS↓
Hu et al. (2022)	China	C57BL/6 mice (7 days old)	NM	T: 6 C: 6	ROP	RSV 50 mg/kg/d, intravitrea, 5d	No treatment	MDA↓, SOD↑
Ji et al. (2021)	China	Male C57BL/6 mice (6–8weeks old)	NM	T: 5 C: 5	I/R	RSV 20 mg/kg/d, IP, 5d	No treatment	RGCs↑ A-Wave↑, B-Wave↑, inner retina thickness↑
Pang et al. (2020)	China	Male SD rats (2–3 months old)	NM	T: 3 C: 3	I/R	RSV 25 mg/kg/d, IP, 3d	Drug solvent	RGCs↑, inner retina thickness↑
Deng et al. (2020)	China	Male SD rats (2–3 months old)	NM	T: 4 C: 4	I/R	RSV 250 mg/kg/d, IP, 3d	No treatment	Total retinal thickness↑, COX-2↓
Cao et al. (2020)	Japan	male C57BL/6 mice (14 weeks old)	25–30	T: 6 C: 6	Glaucoma	RSV 0.004 mg/kg/d, IVT, 21d	No treatment	RGCs↑, ROS↓
Dong et al. (2019)	China	Male Wistar rats (8–10weeks old)	200–250	T: 8 C: 8	DR	RSV 300 mg/kg, OG, 3months	No treatment	Total retinal thickness↑, TNF-α↓, IL-6↓
Chen et al. (2018)	China	Male SD rats (14 weeks old)	200–230	T: 5 C: 5	DR	RSV 0.05 mg/kg/d, IV, 12weeks	No treatment	RGCs↑, TNF-α↓, IL-6
Zhang et al. (2018)	China	Male SD rats (4–6 weeks old)	100–150	T: 6 C: 6	COH	RSV 20 mg/kg/d, OG, 4weeks	Drug solvent	RGCs↑, ROS↓
Luo et al. (2018)	China	SD rats (2–3 months old)	NM	T: 6 C: 6	I/R	RSV 250 mg/kg/d, IP, 3d	No treatment	RGCs↑ Total retinal thickness↑, COX-2↓
Feng et al. (2024)	China	C57BL/6J mice (6–8 weeks old)	NM	T: 4 C: 4	I/R	RSV 20 mg/kg/d, IP, 3d	No treatment	RGCs↑ A-Wave↑, B-Wave↑
Seong et al. (2017)	Korea	Male C57BL⁄6J mice (8 weeks old)	20–25	T: 7 C: 7	I/R	RSV 20 mg/kg/d, IP, 5d	No treatment	RGCs↑, inner retina thickness↑
Zeng et al. (2015)	China	SD rats (14 weeks old)	180 ± 20	T: 5 C: 5	DR	RSV 10 mg/kg/d, OG, 7months	No treatment	B-Wave↑
Razali et al. (2016)	Malaysia	SD rats	NM	T: 24 C: 24	COH	RSV 0.8 mg/kg/d, IVT, 3weeks	Drug solvent	SOD↑, inner retina thickness↑
Pirhan et al. (2015)	Turkey	Wistar rats (adult)	367 ± 12	T: 12 C: 12	Glaucoma	RSV 10 mg/kg, IP, 6weeks	No treatment	RGCs↑
Ghadiri Soufi et al. (2015)	Iran	Male Wistar rats (12 weeks old)	320–350	T: 6 C: 6	DR	RSV 5 mg/kg/d, OG, 4months	No treatment	RGCs↑, COX-2↓, TNF-α↓, IL-6↓
P. Vin et al. (2013)	United States	Male SD rats (adult)	200	T: 6 C: 6	COH	RSV 30 mg/kg/d, IP, 5d	Drug solvent	A- Wave↑ B- Wave↑
Huang et al. (2013)	United States	Male Brown Norway rats	300–450	T: 6 C: 6	Glaucoma	RSV 20 mg/kg/d, IP, 3d	Drug solvent	RGCs↑
Soufi et al. (2012)	Iran	Wistar rats	320–350	T: 6 C: 6	DR	RSV 5 mg/kg/d, OG, 4months	No treatment	SOD↑, TNF-α↓, IL-6↓
Nguyen et al. (2022)	China	SD rats	250–400	T: 6 C: 6	AMD	RSV 0.01 mg/kg/d, IVT, 2months	No treatment	ROS↓, IL-6↓

Abbreviations: T, treatment group; C, control group; NM, not mentioned; RSV, resveratrol; RGCs, retinal ganglion cells; OG, oral gavage; IP, intraperitoneal injections; IV, intravenous injection; IVT, intravitreal injection; DR, diabetic retinopathy; ROP, retinopathy of prematurity; I/R, Ischemia-reperfusion; COH, chronic ocular hypertension; AMD, age-related macular degeneration; COX-2, cyclooxygenase-2; TNF-α, tumour necrosis factor-α; IL-6, interleukin-6; ROS, reactive oxygen species.

### 3.3 Quality evaluation

**FIGURE 2:**
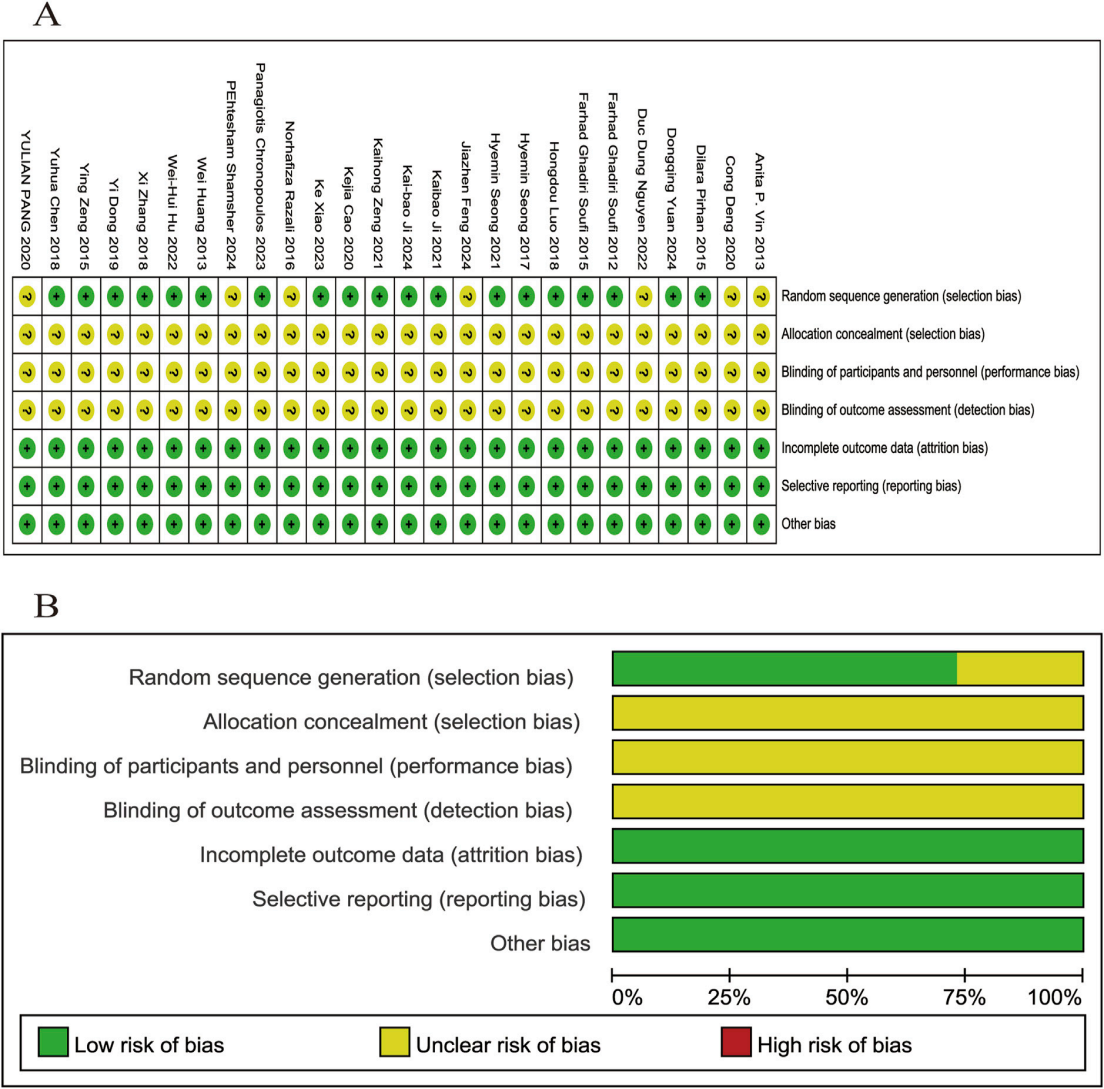
Evaluation of literature quality outcomes derived from SYRCLE’s Risk of Bias utilizing the Cochrane tool. **(A)** Risk of bias summary: the review authors’ assessments of each risk of bias item for each included study; **(B)** Risk of bias graph: review authors’ judgments about each risk of bias item displayed as a percentage for all included studies.

A total of 26 relevant articles were systematically and comprehensively evaluated. While all studies mentioned randomization, 7 did not specify the randomization methods used. Information regarding allocation concealment, blinding of participants and personnel, and blinding of outcome assessment was not provided in any of the studies, resulting in an overall classification of “unclear” for these domains. None of the studies mentioned any other bias, leading to a classification of “low risk” across all studies. The SYRCLE’s risk of bias evaluation is depicted in Figure 2.

### 3.4 Meta analysis of primary outcomes

#### 3.4.1 The number of RGCs

**FIGURE 3:**
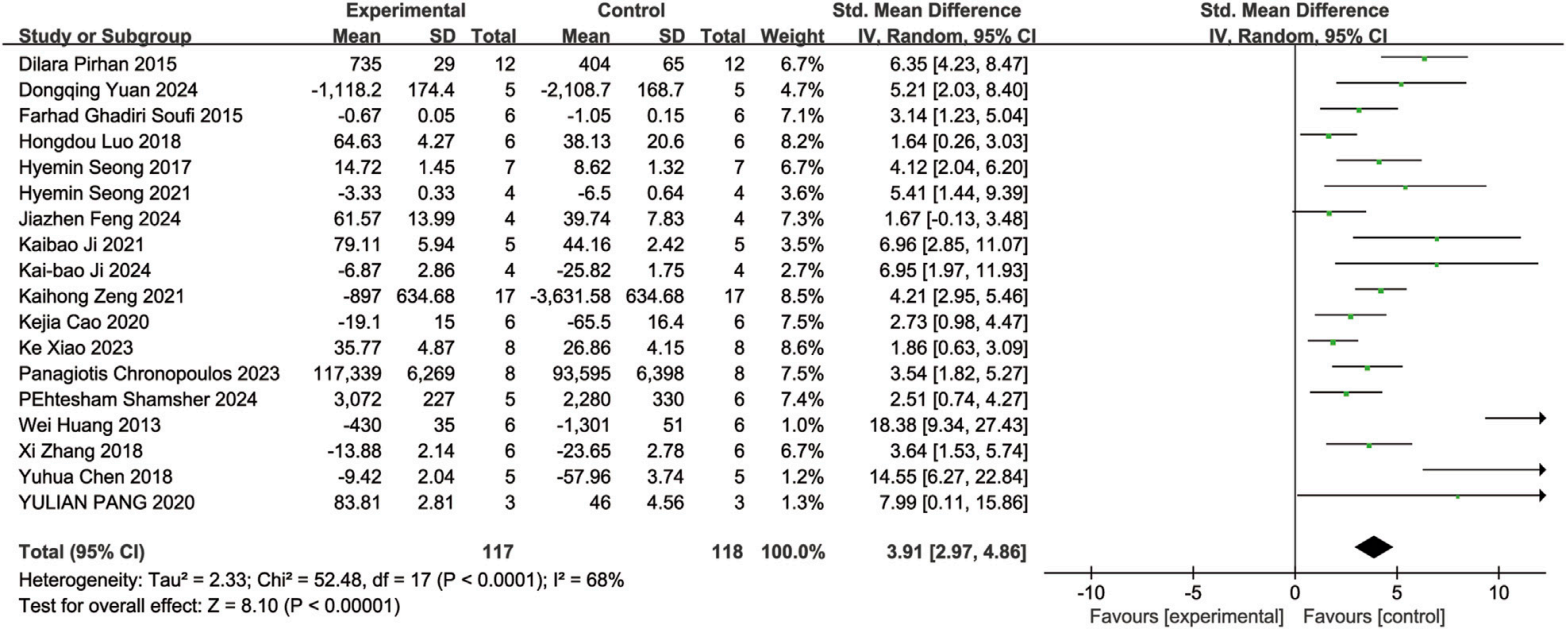
Forest plot of RGCs.

Eighteen of the included studies (Huang et al., 2013; Ghadiri Soufi et al., 2015; Pirhan et al., 2015; Seong et al., 2017; Chen et al., 2018; Luo et al., 2018; Zhang et al., 2018; Cao et al., 2020; Pang et al., 2020; Ji et al., 2021; Seong et al., 2021; Zeng et al., 2021; Chronopoulos et al., 2023; Xiao et al., 2023; Feng et al., 2024; Ji et al., 2024; Shamsher et al., 2024; Yuan et al., 2024) reported the number of RGCs in animal models with retinal disease (experimental group, n = 117; control group, n = 118). The results showed that resveratrol significantly increased the number of RGCs in the retina when compared to the control group (SMD = 3.91, 95% Cl = [2.97, 4.86], *p <* 0.00001) (Figure 3).

**FIGURE 4:**
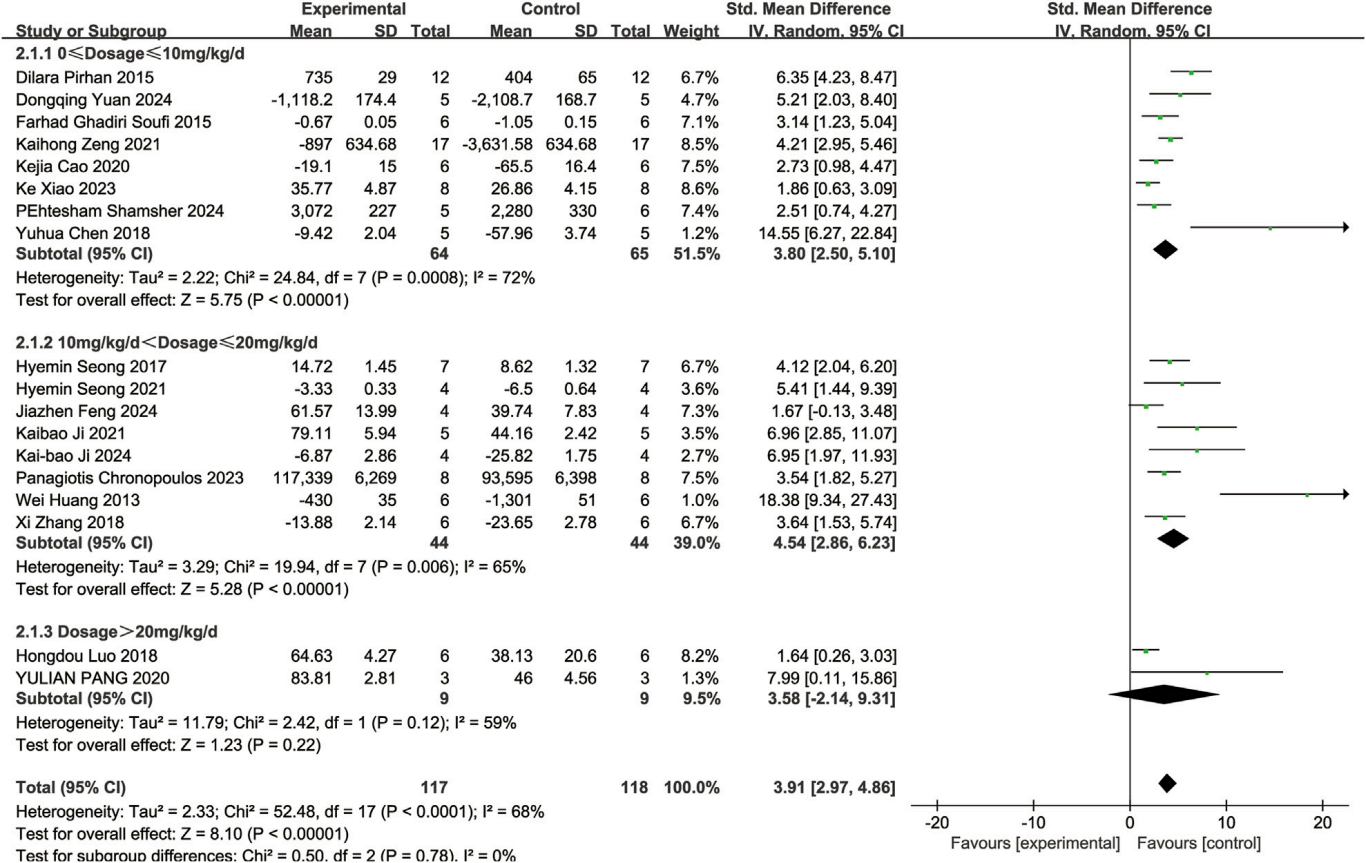
Subgroup analysis of RGCs based on dosage.

A subgroup analysis conducted according to dosage categories (0 mg/kg/d ≤ dosage ≤ 10 mg/kg/d, 10 mg/kg/d < dosage ≤ 20 mg/kg/d, and dosage > 20 mg/kg/d) revealed the following results: for 0 mg/kg/d ≤ dosage ≤ 10 mg/kg/d (SMD = 3.80, 95%Cl = [2.50, 5.10], *p <* 0.00001), for 10 mg/kg/d < dosage ≤ 20 mg/kg/d (SMD = 4.54, 95%Cl = [2.86, 6.23], *p <* 0.00001), and for dosage > 20 mg/kg/d (SMD = 3.58, 95%Cl = [−2.14, 9.31], *p =* 0.22) (Figure 4). Dose-based subgroup analysis showed that although there was significant heterogeneity, different doses of resveratrol could significantly increase the number of RGCs when compared to the control group (SMD = 3.91, 95%Cl = [2.97, 4.86], *p <* 0.00001). However, when comparing the different dosage groups (low, medium, and high doses) with each other, no significant difference was observed in their ability to increase the number of RGCs (*p =* 0.78).

#### 3.4.2 Markers of oxidative stress

**FIGURE 5:**
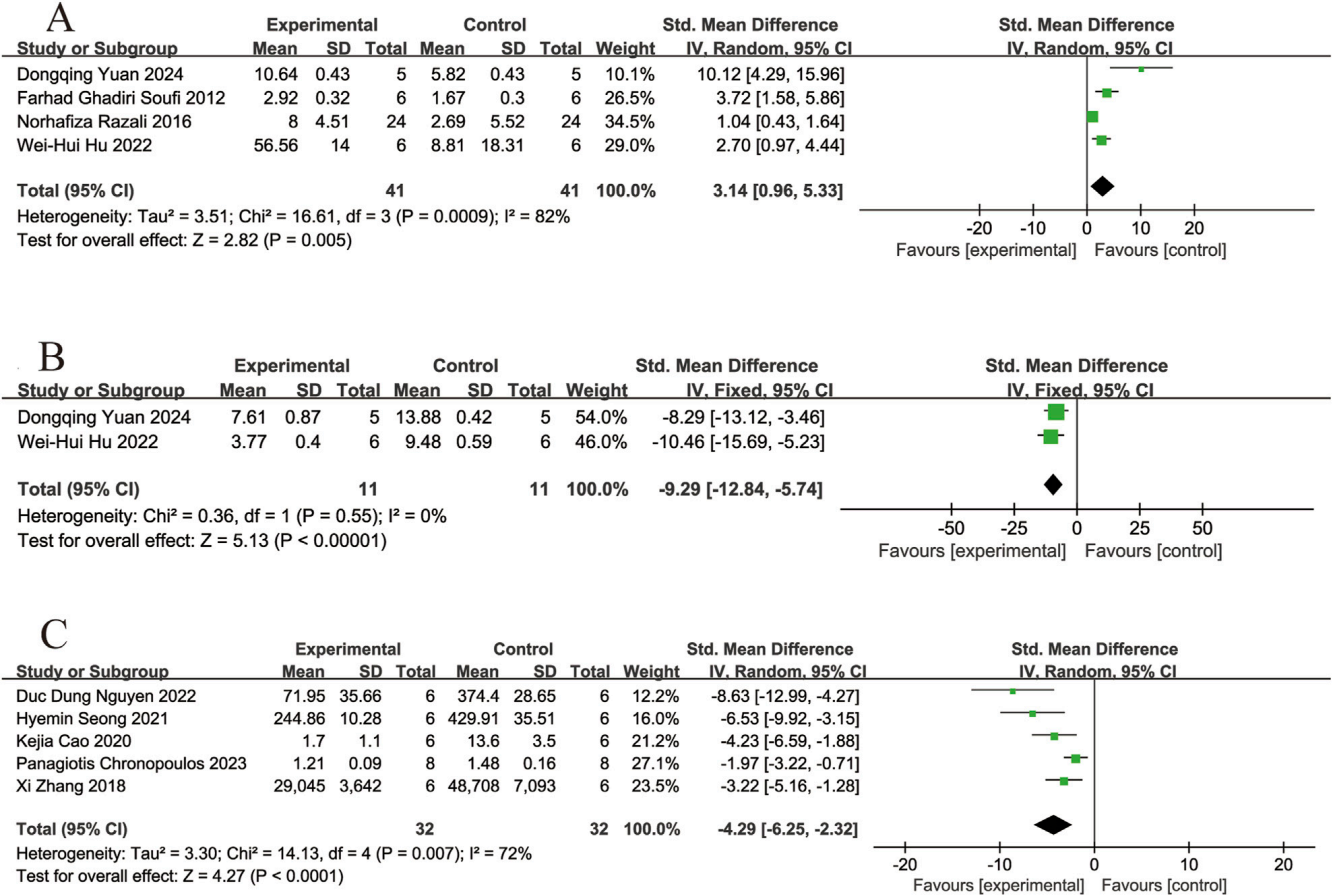
Forest plot. **(A)** The levels of superoxide dismutase; **(B)** The levels of malondialdehyde; **(C)** The levels of reactive oxygen species.

Four of the included studies (Soufi et al., 2012; Razali et al., 2016; Hu et al., 2022; Yuan et al., 2024) reported the effects of resveratrol on SOD activity in animal models with retinal disease (experimental group, n = 41; control group, n = 41). The results showed that resveratrol led to a significant increase in SOD activity in the retina when compared to the control group (SMD = 3.14, 95% Cl = [0.96, 5.33], *p =* 0.005) (Figure 5A).

Two of the included studies (Hu et al., 2022; Yuan et al., 2024) reported the effects of resveratrol on MDA levels in animal models with retinal disease (experimental group, n = 11; control group, n = 11). The results showed that resveratrol significantly reduced the MDA levels in the retina compared with the control group (SMD = −9.29, 95% Cl = [−12.84, −5.74], *p <* 0.00001) (Figure 5B).

Five of the included studies (Zhang et al., 2018; Cao et al., 2020; Seong et al., 2021; Nguyen et al., 2022; Chronopoulos et al., 2023) reported the effects of resveratrol on ROS levels in animal models with retinal disease (experimental group, n = 32; control group, n = 32). The results showed that resveratrol led to a significant reduced in ROS levels in the retina when compared to the control group (SMD = −4.29, 95% Cl = [−6.25, −2.32], *p <* 0.0001) (Figure 5C).

#### 3.4.3 Markers of inflammation

**FIGURE 6:**
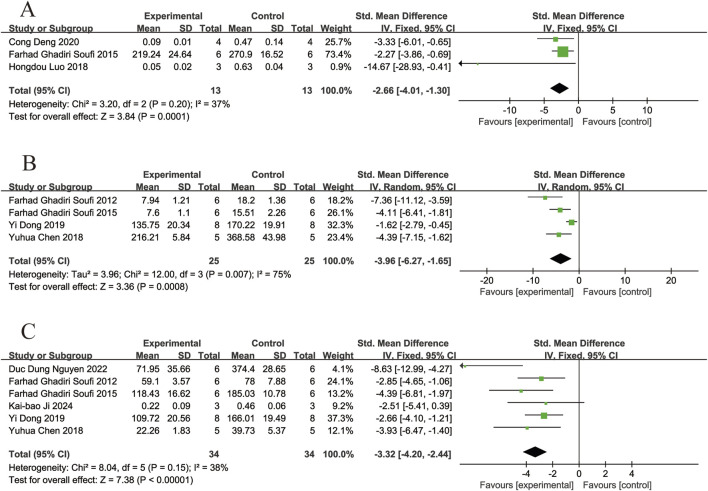
Forest plot. **(A)** The levels of cyclooxygenase-2; **(B)** The levels of tumor necrosis factor-α; **(C)** The levels of interleukin-6.

Three of the included studies (Ghadiri Soufi et al., 2015; Luo et al., 2018; Deng et al., 2020) reported the effects of resveratrol on COX-2 levels in animal models with retinal disease (experimental group, n = 13; control group, n = 13). The results showed that resveratrol led to a significant reduced in COX-2 levels in the retina when compared to the control group (SMD = −2.66, 95% Cl = [−4.01, −1.30], *p =*0.0001) (Figure 6A).

Four of the included studies (Soufi et al., 2012; Ghadiri Soufi et al., 2015; Chen et al., 2018; Dong et al., 2019) reported the effects of resveratrol on TNF-α levels in animal models with retinal disease (experimental group, n = 25; control group, n = 25). The results showed that resveratrol led to a significant reduced in TNF-α levels in the retina when compared to the control group (SMD = −3.96,95% Cl = [−6.27, −1.65], *p =* 0.0008) (Figure 6B).

Six of the included studies (Soufi et al., 2012; Ghadiri Soufi et al., 2015; Chen et al., 2018; Dong et al., 2019; Nguyen et al., 2022; Ji et al., 2024) reported the effects of resveratrol on IL-6 levels in animal models with retinal disease (experimental group, n = 34; control group, n = 34). The results showed that resveratrol led to a significant reduced in IL-6 levels in the retina when compared to the control group (SMD = −3.32, 95% Cl = [−4.20, −2.44], *p <* 0.00001) (Figure 6C).

#### 3.4.4 Electroretinography

**FIGURE 7:**
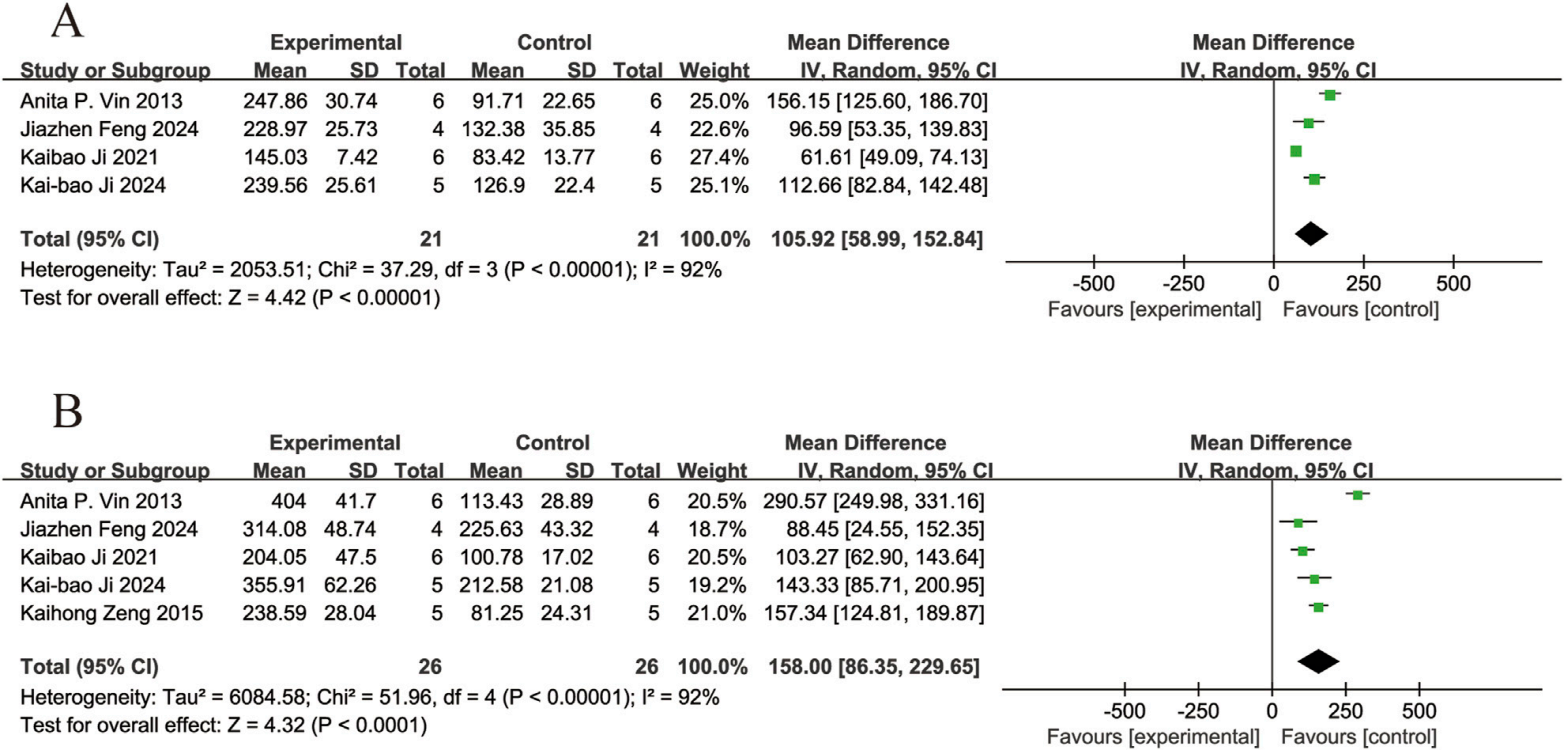
Forest plot. **(A)** The amplitudes of A-wave; **(B)** The amplitudes of B-wave.

Four of the included studies (Vin et al., 2013; Ji et al., 2021; 2024; Feng et al., 2024) reported the effects of resveratrol on A-wave amplitudes in animal models with retinal disease (experimental group, n = 21; control group, n = 21). The results showed that resveratrol significantly increased the A-wave amplitudes in the retina compared with the control group (MD = 105.92, 95% Cl = [58.99, 152.84], *p <* 0.00001) (Figure 7A).

Five of the included studies (Vin et al., 2013; Zeng et al., 2015; Ji et al., 2021; 2024; Feng et al., 2024) reported the effects of resveratrol on B-wave amplitudes in animal models with retinal disease (experimental group, n = 26; control group, n = 26). The results showed that resveratrol significantly increased the B-wave amplitudes in the retina compared with the control group (MD = 158.00, 95% Cl = [86.35, 229.65], *p <* 0.0001) (Figure 7B).

#### 3.4.5 Retinal thickness

**FIGURE 8:**
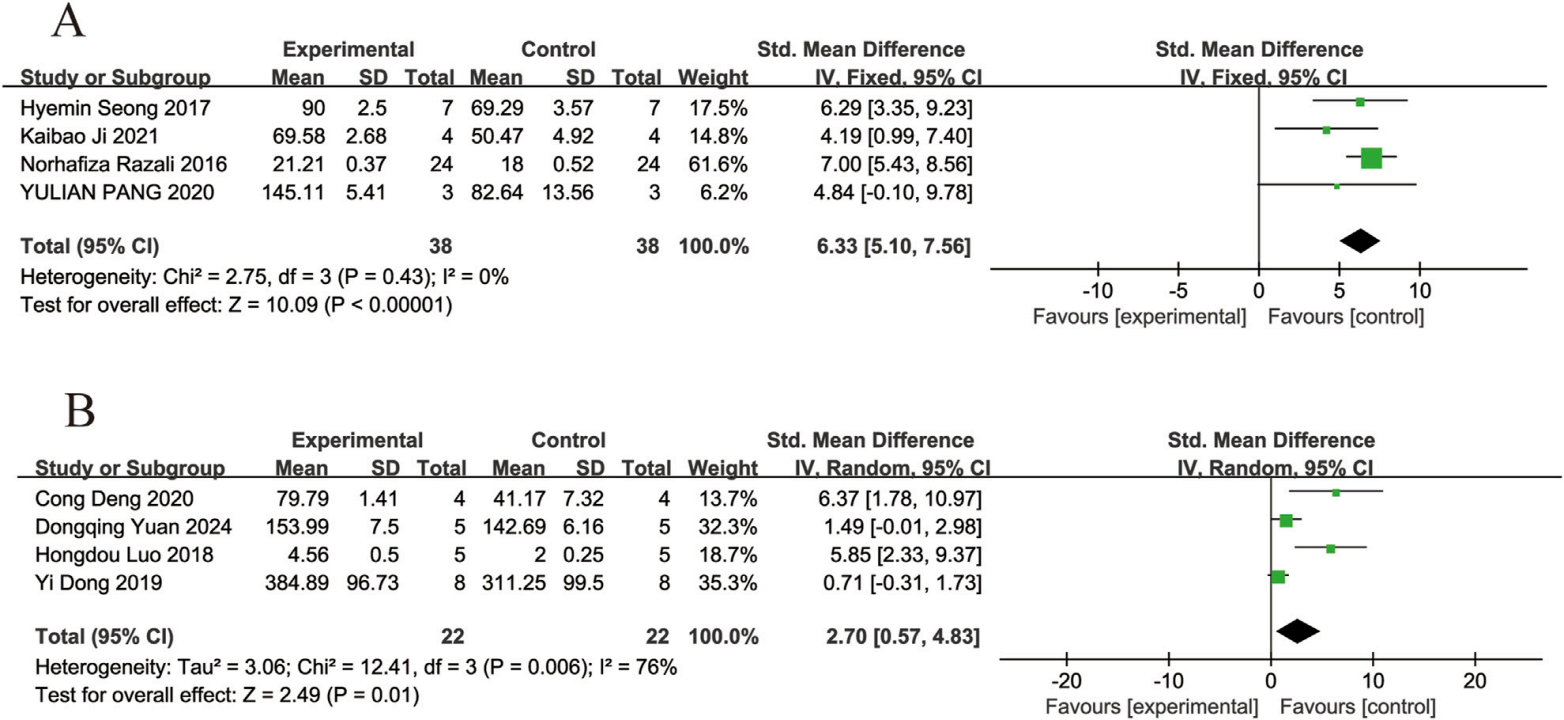
Forest plot. **(A)** The thickness of inner retinal; **(B)** The thickness of total retinal.

Four of the included studies (Razali et al., 2016; Seong et al., 2017; Pang et al., 2020; Ji et al., 2021) reported the effects of resveratrol on inner retinal thickness in animal models with retinal disease (experimental group, n = 38; control group, n = 38). The results showed that resveratrol significantly increased inner retinal thickness compared with the control group (SMD = 6.33, 95% Cl = [5.10, 7.56], *p <* 0.00001) (Figure 8A).

Four of the included studies (Luo et al., 2018; Dong et al., 2019; Deng et al., 2020; Yuan et al., 2024) reported the effects of resveratrol on total retinal thickness in animal models with retinal disease (experimental group, n = 22; control group, n = 22). The results showed that resveratrol significantly increased the total retinal thickness in the retina compared with the control group (SMD = 2.70, 95% Cl = [0.57, 4.83], *p =* 0.01) (Figure 8B).

### 3.5 Publication bias analysis

**FIGURE 9:**
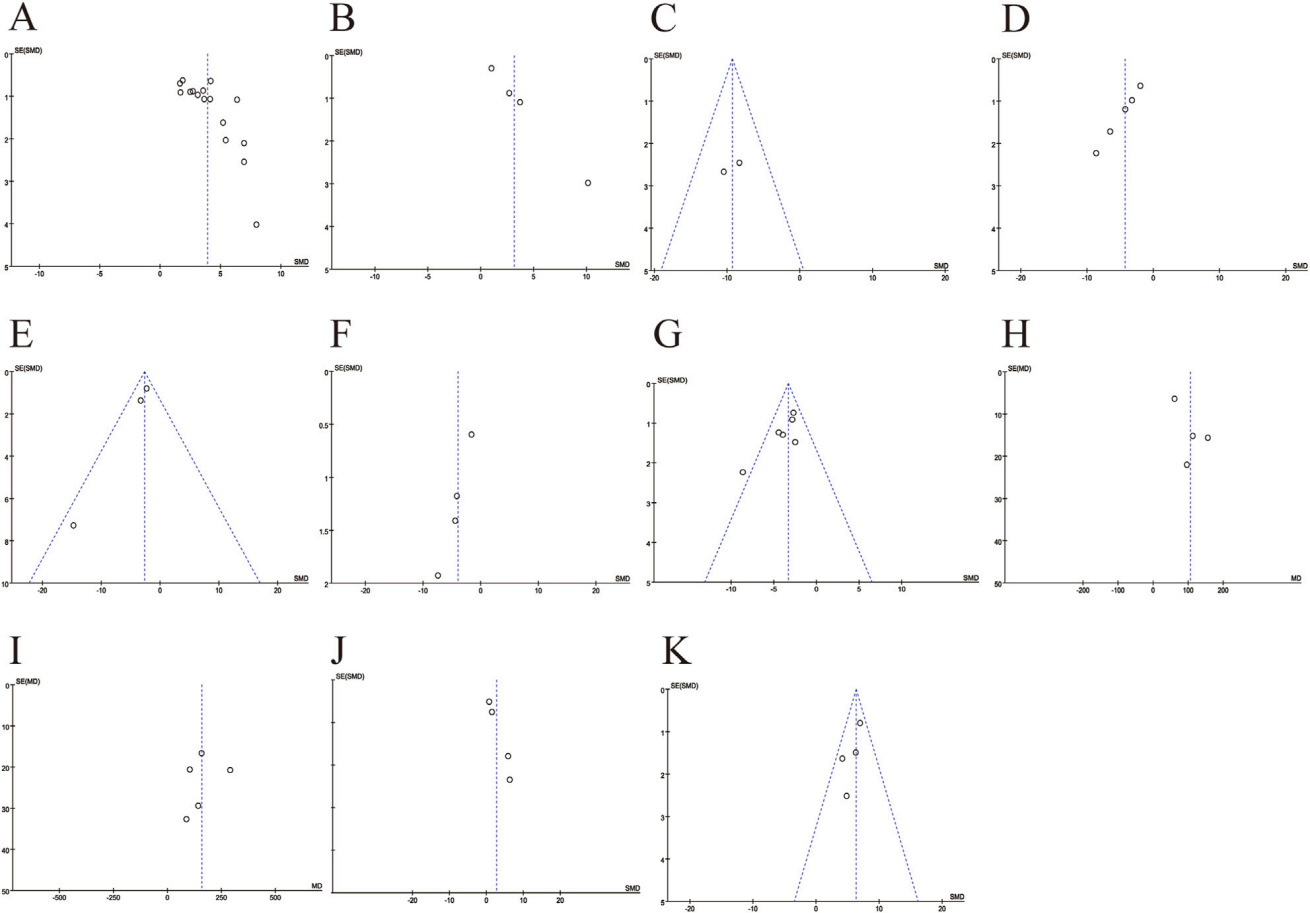
Funnel plots for evaluating publication bias. **(A)** The number of retinal ganglion cells; **(B)** The levels of superoxide dismutase; **(C)** The levels of malondialdehyde; **(D)** The levels of reactive oxygen species; **(E)** The levels of cyclooxygenase-2; **(F)** The levels of tumor necrosis factor-α; **(G)** The levels of interleukin-6; **(H)** The amplitudes of A-wave; **(I)** The amplitudes of B-wave; **(J)** The thickness of total retinal; **(K)** The thickness of inner retinal.

To assess publication bias in the meta-analysis of the outcome indicators (Figures 9A–K), the results showed asymmetry in the funnel plot. The scatter of the funnel plot of the number of RGCs (A), SOD (B), ROS(D), COX-2 (E), TNF-α (F) and total retinal thickness (J) deviated from the axis of symmetry, which may be related to the small sample study, the type of animal, and the difference in detection methods. The scatter distributions of IL-6 (G), A-wave (H), B-wave (I), and inner retinal thickness (K) were relatively scattered with weak asymmetry. Egger’s test were used to quantitatively analyze publication bias (Table 3). The results indicate the presence of publication bias for the number of RGCs, SOD, ROS, COX-2, TNF-α and total retinal thickness (*p <* 0.05).

**TABLE 3:** Egger’s test for evaluating publication bias.

Outcome	Egger’s test
	*p*-Value
The number of RGCs	0.000
SOD	0.003
MDA	—
ROS	0.000
COX-2	0.003
TNF-α	0.008
IL-6	0.059
A-wave	0.195
B-wave	0.571
Inner retinal thickness	0.181
Total retinal thickness	0.010

### 3.6 Sensitivity analysis

**FIGURE 10:**
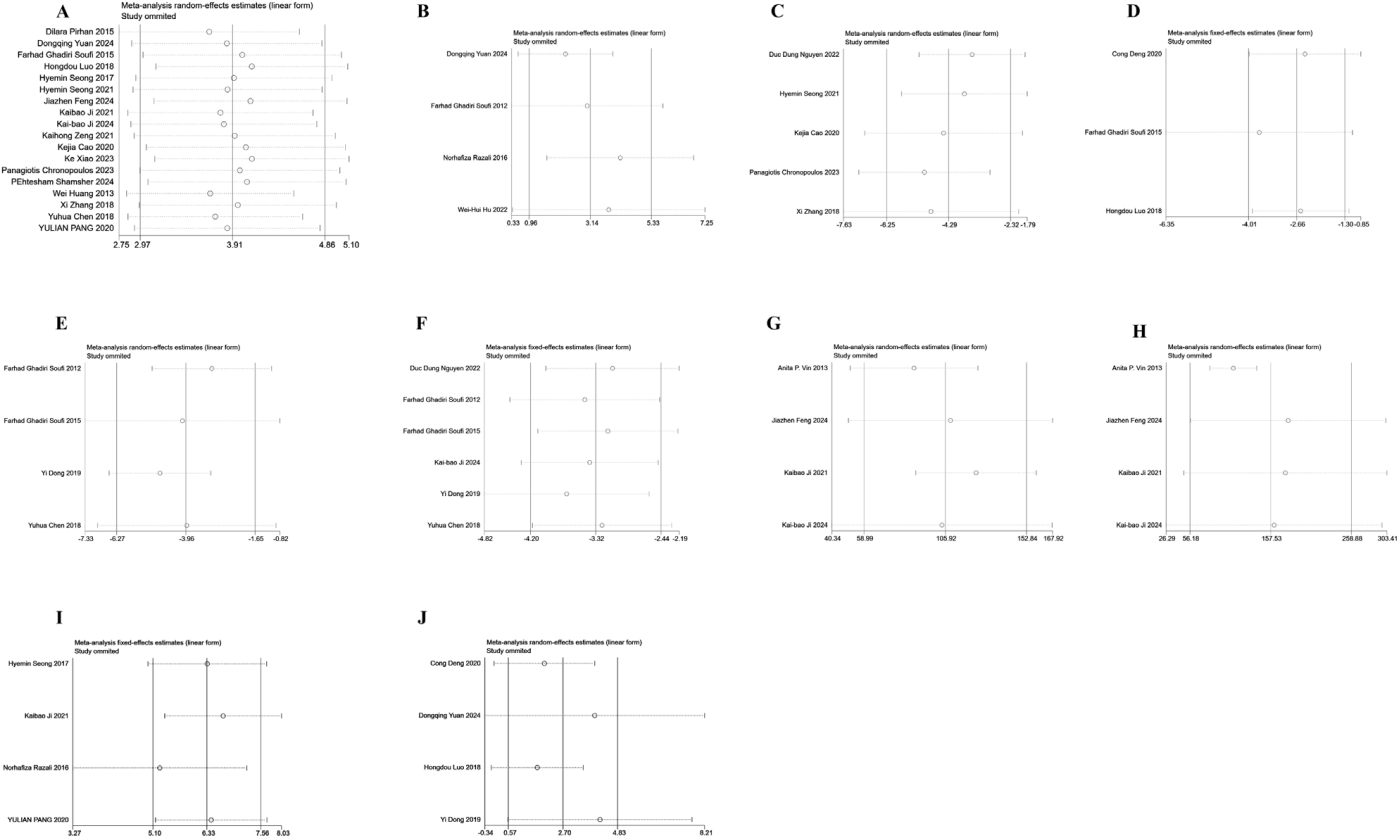
Sensitivity analysis chart. **(A)** The number of retinal ganglion cells; **(B)** The levels of superoxide dismutase; **(C)** The levels of reactive oxygen species; **(D)** The levels of cyclooxygenase-2; **(E)** The levels of tumor necrosis factor-α; **(F)** The levels of interleukin-6; **(G)** The amplitudes of A-wave; **(H)** The amplitudes of B-wave; **(I)** The thickness of inner retinal; **(J)** The thickness of total retinal.

To evaluate the consistency and reliability of the meta-analysis results, Outcome measures included the number of RGCs (A), SOD (B), ROS (C), COX-2 (D), TNF-α (E), IL-6 (F), A-wave amplitudes (G), B-wave amplitudes (H), inner retinal thickness(I), and total retinal thickness (J), sensitivity analysis was performed. By recalculating the pooled effect size after excluding individual studies one by one, the pooled effect size of each of the above indicators was not significantly changed by the exclusion of individual studies. The conclusion of this meta-analysis is robust (Figures 10A–J).

## 4 Discussion

This systematic review mainly investigated the therapeutic effects of resveratrol on retinal diseases in animal models from multiple aspects, such as retinal neuroprotection, structural function, oxidative stress,and inflammatory factors. The findings demonstrated a beneficial impact of resveratrol in enhancing the evaluated indices of retinal injury post-treatment. Retinal diseases can result in reduced RGC numbers, consequently affecting visual function (Yuan et al., 2024). Excessive generation of ROS can lead to oxidative stress. SOD is the primary enzyme for scavenging oxygen free radicals in biological systems, whereas MDA serves as a crucial marker reflecting the extent of damage caused by free radicals. In retinal diseases, a reduction in SOD levels and an elevation in MDA and ROS levels signify heightened oxidative stress, impacting the standard function and metabolism of retinal cells, consequently contributing to the occurrence of retinal diseases (Dziedziak et al., 2021). As an inflammatory marker, TNF-α mainly causes retinal damage through inflammatory and apoptotic pathways. The level of IL-6 is related to the severity of retinal diseases, while COX2 participates in disease progression by regulating inflammation and angiogenesis. Therefore, these factors and their signaling pathways may be important targets for the treatment of retinal diseases. The A-wave in the electroretinogram predominantly mirrors the functionality of retinal photoreceptors, while the B-wave indicates the signal transmission process between the inner retinal nerve cells. Therefore, abnormal changes in the amplitudes of A and B waves can reveal different mechanisms of retinal damage (Xiao et al., 2023). There is a significant correlation between changes in retinal thickness and visual function, with variations in inner retinal thickness serving as an early indicator of retinopathy (Chronopoulos et al., 2023; Feng et al., 2024). Therefore, we collected and analyzed the published high-quality experimental data to investigate the application of resveratrol on retinal diseases.

This study showed that resveratrol significantly increased the number of RGCs in animal models treating retinal diseases. Dose-based subgroup analyses showed similar results, although high heterogeneity was observed at lower doses. Researcher have studied the neuroprotective effect of resveratrol on RGC survival in ischemic and reperfusion retinal injury (I/R injury). Their findings revealed that resveratrol regulates a variety of signaling pathways, including SIRT1/NF-κB axis and SIRT1-JNK pathway, by activating SIRT1, thereby decreasing the programmed cell death and axonal deterioration of retinal ganglion cells (Wu et al., 2020). The study demonstrated that upon activation of the Nrf2 signaling pathway, resveratrol can significantly reduce the expression of apoptosis protein Caspase-3. Caspase-3 serves as a pivotal effector protein in the apoptosis cascade, and its reduced expression signifies a decrease in apoptosis, thereby exerting a protective role on retinal ganglion cells and improving the pathological process of diabetic retinopathy (Yuan et al., 2024). In conclusion, the current study suggests that resveratrol has a significant neuroprotective effect in the protection of retinal ganglion cells by regulating and reducing protein expression through signaling pathways.

The retina may sustain structural and functional harm as a result of prolonged oxidative stress. The results of this study show that resveratrol can significantly increase the activity of SOD and reduce the levels of MDA and ROS, thereby reducing the damage of the retina caused by oxidative stress. Additionally, although heterogeneity was observed in the SOD and ROS studies, subgroup analysis could not be performed due to the insufficient number of articles. Meanwhile, only two studies examined the MDA index. SOD is considered to be an important antioxidant that safeguards cells by neutralizing free radicals, thereby preserving the oxidative/antioxidant balance in the body during oxidative stress. As an important product in the oxidative damage process, MDA is an important index to measure the degree of oxidative damage (Pisoschi and Pop, 2015). Nrf2 functions as a transcription factor capable of modulating the expression of numerous antioxidant enzymes. There are studies further explored and found that resveratrol can elevate SOD expression by activating the Nrf2 signaling pathway (Wang et al., 2025). Furthermore, through the augmentation of antioxidant enzyme activity such as SOD, resveratrol can effectively clear intracellular ROS, reduce the occurrence of lipid peroxidation, and thus decreas the generation of MDA. In conclusion, resveratrol can effectively protect retinal cells from oxidative stress by regulating the levels of SOD, MDA and ROS. This protective mechanism has significant preventive and management significance for retinal diseases caused by oxidative stress.

The results of this study demonstrate that resveratrol can significantly decrease the levels of inflammatory markers. Resveratrol reduces the expression of matrix metalloproteinases and inhibits the production of IL-6 and TNF-α (Fisher, 2021). Furthermore, it has been shown that resveratrol effectively suppresses NF-κB (nuclear factor-kappa B) signaling by inhibiting NF-κB activity and suppressing the phosphorylation of JAK/STAT signaling pathways. Numerous polyphenols inhibit the expression of COX genes, and it has been established that resveratrol inhibits the activity of COX-1 and COX-2 in a dose-dependent manner (Li et al., 2018). It is reported that resveratrol may further exert neuroprotective effects by activating Peroxisome Proliferator-Activated Receptors (PPARs) and inhibiting the production of NO to resist oxidative stress and inflammatory damage (Yunusoğlu, 2021). These mechanisms of action provide a theoretical basis for the potential application of resveratrol in the treatment of retinal diseases (Wu et al., 2020).

The results of this study show that resveratrol can significantly enhancing the amplitude of A and B waves in electroretinogram. A wave and B wave are waveforms in electroretinogram (ERG), with the A wave predominantly reflecting the functionality of retinal photoreceptors. Resveratrol enhances the A-wave amplitude by safeguarding photoreceptor cells. In particular, under conditions such as light damage or diabetic retinopathy, resveratrol can protect photoreceptor cells and reduce their apoptosis to enhance the a-wave signal intensity (Luo et al., 2018). The B-wave primarily correlates with the function of retinal bipolar cells. Research has demonstrated that resveratrol enhances signal transmission between bipolar cells and retinal ganglion cells by suppressing retinal inflammation and oxidative stress, consequently elevating the B-wave amplitude (Wu et al., 2020). Therefore, resveratrol has a protective effect on the A and B-wave amplitudes in electroretinogram through various mechanisms, effectively enhancing retinal function and showcasing potential application value in the treatment of retinal diseases.

Inner retinal thickness refers to the thickness of each layer of the innermost layer of the retina, encompassing structures such as the ganglion cell layer and the inner core layer. Progressive pathological conditions often lead to a notable reduction in inner retinal thickness, detectable at early stages. Total retinal thickness represents the cumulative thickness of all retinal layers, serving as an indicator of overall retinal health. The outcomes of this study demonstrated a significant enhancement in both inner retinal thickness and total retinal thickness following resveratrol treatment. The study demonstrated that resveratrol mitigates retinal ganglion cell apoptosis by regulating SIRT1-JNK pathway (Luo et al., 2018). Moreover, resveratrol inhibits retinal neuronal cell apoptosis by upregulating Bcl-2 expression and downregulating Caspase-3 expression, thereby preserving inner retinal structure. Furthermore, resveratrol diminishes retinal damage through the inhibition of inflammatory factors, specifically TNF-α and IL-6,that helps preserve both the overall structure and thickness of the retina. Therefore, resveratrol exerts a protective influence on inner retinal thickness and total retinal thickness through diverse mechanisms, effectively enhancing retinal structure, which has potential application value in the treatment of retinal diseases.

This study represents the first comprehensive investigation into the impact of resveratrol on animal models of retinal diseases. To provide more reliable evidence for the protection of resveratrol on retinal disease models by multidimensional evaluation of funnel plot, Egger’s test and sensitivity analysis. Despite the meticulous screening and assessment, there are still deficiencies. Firstly, detailed information regarding the characteristics of resveratrol, such as content and properties, was not provided in the study, potentially introducing certain discrepancies in the results. Secondly, the imbalance observed in Egger’s test and the funnel plot suggests the presence of publication bias, which may affect the interpretation of the results. The high heterogeneity may result from different study designs, including differences in animal models, methods, doses, and durations. It is recommended that future studies consider these potential sources of heterogeneity to improve the consistency of study designs. Finally, the studies that were included lacked descriptions of assignment concealment, randomization of animals during experiments, and blinding of investigators.

## 5 Conclusion

This meta-analysis included 26 articles that summarized resveratrol’s influence on retinal diseases while also offering significant preclinical mechanistic studies in terms of neuroprotection, structural function, and oxidative inflammatory markers. Our meta-analysis results show that resveratrol affects several markers of retinal diseases, implying positive effects on animal models of retinal diseases and establishing it as a prospective adjuvant that can improve retinal diseases. Nonetheless, given the heterogeneity, potential for bias, and other limitations of the included studies, more high-quality studies are needed to confirm resveratrol’s effectiveness in treating retinal diseases.

## Data Availability

The original contributions presented in the study are included in the article/Supplementary Material, further inquiries can be directed to the corresponding author.
