# Supplementary material for: A comprehensive and systematic review on resveratrol supplementation as a promising candidate for the retinal disease: a focus on mechanisms of action from preclinical studies
Source: Front Pharmacol. 2025 Jul 11;16:1615910. doi: 10.3389/fphar.2025.1615910 (PMC12289659; doi:10.3389/fphar.2025.1615910)
Supplement: Supplementary file 1 [file Table1.doc]

| Search | PUBMED 176 |
| --- | --- |
| #1 | resveratrol[MeSH Terms] |
| #2 | (((((((((((((((resveratrol) OR (3,4',5-Stilbenetriol)) OR (3,5,4'-Trihydroxystilbene)) OR (3,4',5-Trihydroxystilbene)) OR (trans-Resveratrol)) OR (trans Resveratrol)) OR (Resveratrol-3-sulfate)) OR (Resveratrol 3 sulfate)) OR (SRT 501)) OR (SRT-501)) OR (SRT501)) OR (cis-Resveratrol)) OR (cis Resveratrol)) OR (Resveratrol, (Z)-)) OR (trans-Resveratrol-3-O-sulfate)) OR (trans Resveratrol 3 O sulfate) |
| #3 | (#1) OR (#2) |
| #4 | retina[MeSH Terms] |
| #5 | (retina) OR (Ora Serrata) |
| #6 | (#4) OR (#5) |
| #7 | (#3) AND (#6) |
|  |  |
|  | Embase 101 |
| #1 | resveratrol:ab,ti OR '3,4,5 stilbenetriol':ab,ti OR '3,5,4 trihydroxystilbene':ab,ti OR '3,4,5 trihydroxystilbene':ab,ti OR 'trans resveratrol':ab,ti OR 'trans resveratrol':ab,ti OR 'resveratrol 3 sulfate':ab,ti OR 'resveratrol 3 sulfate':ab,ti OR 'srt 501':ab,ti OR srt501:ab,ti OR 'cis resveratrol':ab,ti OR 'cis resveratrol':ab,ti OR (resveratrol,:ab,ti AND z:ab,ti AND -:ab,ti) OR 'trans resveratrol 3 o sulfate':ab,ti OR 'trans resveratrol 3 o sulfate':ab,ti |
| #2 | retina:ab,ti OR 'ora serrata':ab,ti |
| #3 | #1 AND #2 |
|  |  |
|  | Web of Science 151 |
| #1 | (((((((((((((((ALL=(resveratrol )) OR ALL=(3,4',5-Stilbenetriol)) OR ALL=(3,5,4'-Trihydroxystilbene)) OR ALL=(3,4',5-Trihydroxystilbene)) OR ALL=(trans-Resveratrol )) OR ALL=(trans Resveratrol)) OR ALL=(Resveratrol-3-sulfate)) OR ALL=(Resveratrol 3 sulfate)) OR ALL=(SRT 501)) OR ALL=(SRT-501)) OR ALL=(SRT501)) OR ALL=(cis-Resveratrol)) OR ALL=(cis Resveratrol)) OR ALL=(Resveratrol, (Z)-)) OR ALL=(trans-Resveratrol-3-O-sulfate)) OR ALL=(trans Resveratrol 3 O sulfate) |
| #2 | (ALL=(retina )) OR ALL=(Ora Serrata) |
| #3 | #2 AND #1 |
|  |  |
|  | Cochrane 6 |
|  | 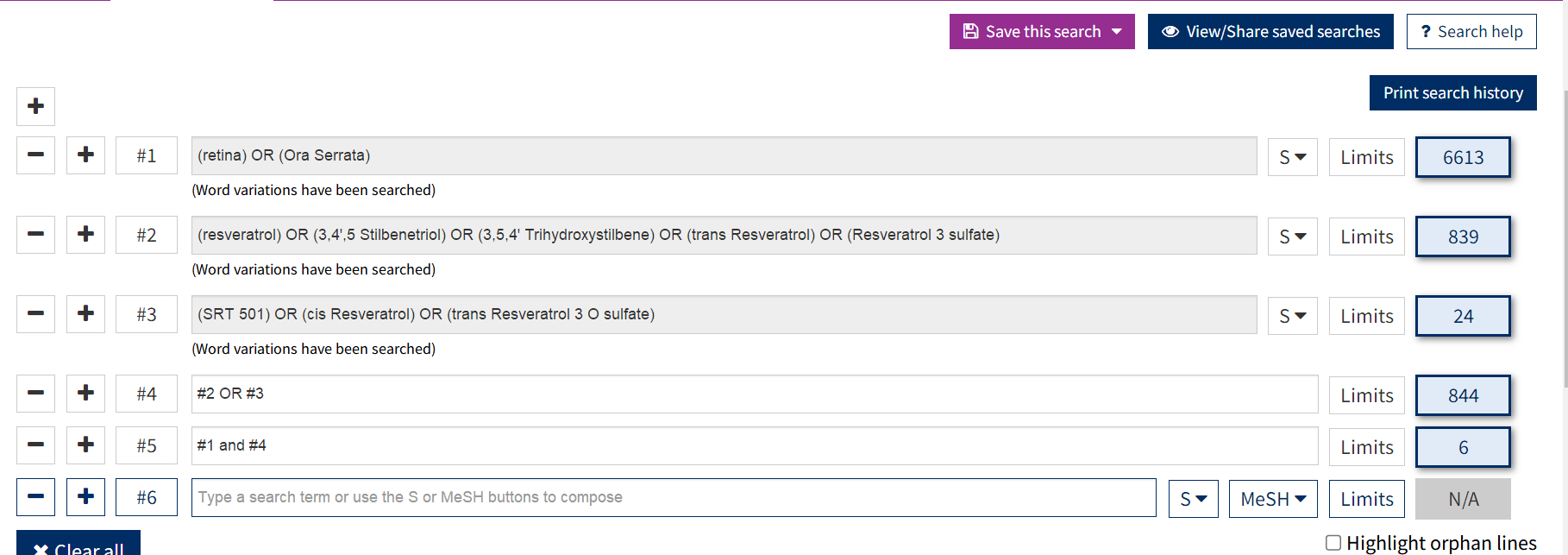 |
|  | OVID 116 |
|  | 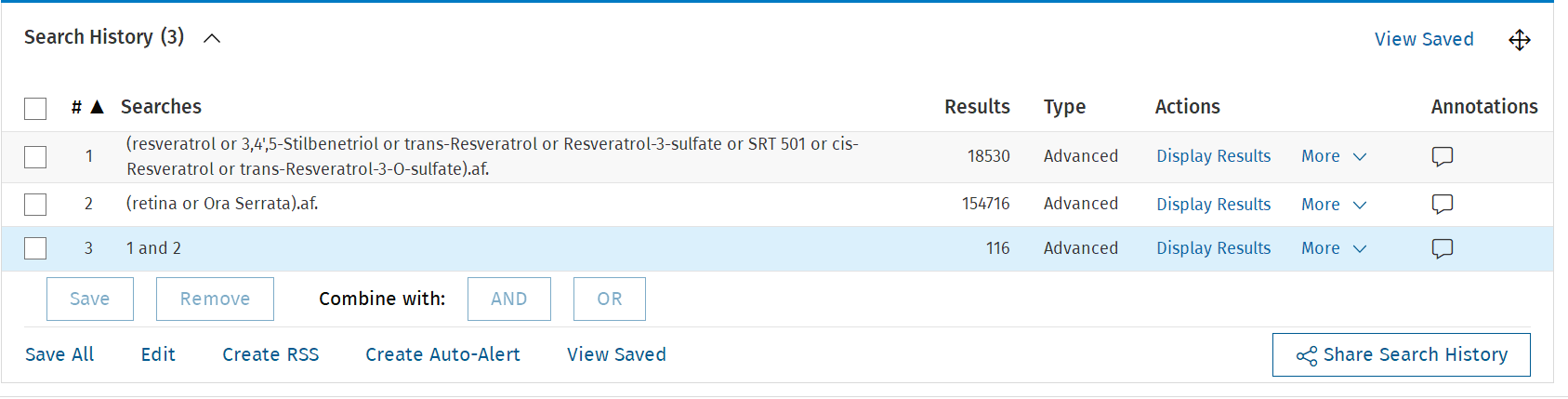 |
|  |  |
